# Multiscale Brain Aging in the Context of Neurodegeneration and Alzheimer's Disease

**DOI:** 10.1093/geroni/igab046.1435

**Published:** 2021-12-17

**Authors:** Kyra Thrush, Yaroslav Markov

## Abstract

The brain, with a diverse array of specialized cells, regional substructures, and a relatively isolated microenvironment, represents a uniquely challenging organ system for aging research. The brain can experience physical trauma, interact with the periphery, and is responsible for cognitive and behavioral modifications that can feed back into the molecular processes of aging both within and external to the brain. Advances to our understanding and ability to intervene in the complexity that personifies brain aging and associated neurodegeneration will require integrated, multiscale approaches operating in tandem. Therefore, we have organized this symposium to highlight promising new approaches to study brain aging through the lens of multiple biological levels of organization. We will provide insight not only into normal brain aging, but will also suggest key spurious processes that may drive neurodegeneration and functional decline.

